# Advancing Post-Stroke Depression Research: Insights from Murine Models and Behavioral Analyses

**DOI:** 10.3390/life14091110

**Published:** 2024-09-03

**Authors:** Mădălina Iuliana Mușat, Bogdan Cătălin, Michael Hadjiargyrou, Aurel Popa-Wagner, Andrei Greșiță

**Affiliations:** 1 Experimental Research Centre for Normal and Pathological Aging, University of Medicine and Pharmacy of Craiova, 200349 Craiova, Romania; madalina.musat3@gmail.com (M.I.M.); aurel.popa-wagner@geriatrics-healthyageing.com (A.P.-W.);; 2 Department of Biological and Chemical Sciences, New York Institute of Technology, Old Westbury, NY 11568, USA; 3 Department of Neurology, Vascular Neurology and Dementia, University of Medicine Essen, 45122 Essen, Germany; 4 Department of Biomedical Sciences, New York Institute of Technology, Old Westbury, NY 11568, USA

**Keywords:** post-stroke depression, behavioral tests, cognition, social activity, motor function, antidepressants, murine models of depression

## Abstract

Post-stroke depression (PSD) represents a significant neuropsychiatric complication that affects between 39% and 52% of stroke survivors, leading to impaired recovery, decreased quality of life, and increased mortality. This comprehensive review synthesizes our current knowledge of PSD, encompassing its epidemiology, risk factors, underlying neurochemical mechanisms, and the existing tools for preclinical investigation, including animal models and behavioral analyses. Despite the high prevalence and severe impact of PSD, challenges persist in accurately modeling its complex symptomatology in preclinical settings, underscoring the need for robust and valid animal models to better understand and treat PSD. This review also highlights the multidimensional nature of PSD, where both biological and psychosocial factors interplay to influence its onset and course. Further, we examine the efficacy and limitations of the current animal models in mimicking the human PSD condition, along with behavioral tests used to evaluate depressive-like behaviors in rodents. This review also sets a new precedent by integrating the latest findings across multidisciplinary studies, thereby offering a unique and comprehensive perspective of existing knowledge. Finally, the development of more sophisticated models that closely replicate the clinical features of PSD is crucial in order to advance translational research and facilitate the discovery of future effective therapies.

## 1. Background

Studies estimate that annually, 15 million people are affected by a stroke worldwide. Roughly 5 million people die due to the ischemic event, while another 5 million survivors are permanently disabled [1,2]. Unfortunately, prevention cannot successfully reduce the stroke occurrence rate due to the high number of risk factors and comorbidities linked to its onset [3]. These include the most important factors, cardiovascular disease [4], high body mass index [5], chronic stress [6], and more importantly, aging [7]. Current therapeutic approaches are mostly focused on limiting the long-term multiple medical conditions of stroke survivors as well as the overall burden on the healthcare system and society [8]. 

Following stroke, motor complications, such as hemiparesis [9], hemiplegia, or postural instability [10], significantly challenge patient rehabilitation efforts [11]. Additionally, patients are faced with urinary and bowel incontinence [12], cognitive impairment and dementia [13], depression [14], anxiety [15], fatigue [16], and sleep disorders [17,18], all of which pose a significant challenge for successful diagnosis and treatment. While significant motor [11] and cognitive [19] dysfunction represent long-term consequences of ischemic injury, PSD represents the most frequent and challenging neuropsychiatric complication [20,21]. 

Estimating the exact prevalence of PSD is challenging, particularly due to methodological complexities, such as variations in the timing of patient evaluations after stroke onset and differences in instruments and criteria utilized in experimental settings [21]. However, recent findings suggest that PSD occurs in approximately 18–33% of cases [22,23], with the greatest number of occurrences observed within the first year following the ischemic insult [24]. Also, ~53% of individuals who were depressed within 3 months after stroke experienced persistent depression [21]. These results are concerning, especially because the mortality rate is higher among patients who suffer from PSD [22]. The risk of suicidal death is ~2 times higher for stroke patients compared to the general population [25], and in addition to suicidal ideation [26], cognitive deficits [27], long-term disability [28], and a substantially lower quality of life [29] are additional symptoms in patients who develop PSD.

Thus, it is of paramount importance to understand the underlying mechanisms of this disease and to accurately identify efficient screening tools and therapeutic modalities. However, studying the pathopsychological mechanisms underlying PSD, such as affected cellular plasticity [30], neuroinflammation, and neurodegeneration [31], as well as intrinsic recovery pathways (neurogenesis) [32], requires a highly accurate and performant experimental approach. But, establishing preclinical models can also pose challenges, particularly given the subjective nature of the psychological and physiological PSD symptoms [33]. Researchers have endeavored to create rodent models that capture key aspects of PSD, allowing for the evaluation of behavioral manifestations and underlying neurobiological changes [34,35,36]. One such approach involves inducing focal cerebral ischemia in rodents [37] and simulating the conditions of stroke observed in humans. Following a stroke, protocols for inducing depressive behavior [38] and behavioral tests tailored to assess depressive-like symptoms are used [39] and encompass a wide range of assays designed to evaluate various aspects of depressive behavior, including despair-like behavior [40], anhedonia [41], and alterations in locomotor activity and exploration [42,43]. 

Despite recent progress in PSD-related research, challenges persist in accurately reproducing the multifaceted nature of this condition in in vivo animal models. To advance translational research and the development of new therapeutic treatments for PSD, it is crucial to develop valid animal models that accurately replicate the complex symptomatology of the condition. According to the International Classification of Diseases, 11th Revision (ICD 11) criteria [44], psychiatrists diagnosing depression in patients typically confirm the presence of five or more symptoms from the depressive spectrum (i.e., depressed mood, anhedonia, appetite or weight changes, sleep disturbances, psychomotor agitation or retardation, fatigue, reduced concentration, feelings of worthlessness or excessive guilt, and recurrent thoughts of suicide or death) [45]. Accurate replication of the human condition in these models is crucial for understanding the pathology and to effectively test various therapeutic interventions. Variability in individual responses [46], differences in genetic backgrounds [47,48,49,50,51], and the subjective nature of depressive symptoms [52,53] all present challenges in interpreting the data effectively. Moreover, the environmental and social factors that can influence the onset and progression of PSD in humans [54,55] are difficult to replicate in murine models, limiting the validity and effectiveness of these studies. The complexity of stroke-induced brain injury, which involves not just the neural circuits traditionally associated with mood regulation [56,57] but also the broader neurological disruptions [58], complicates the accuracy of modeling PSD. Additionally, the lack of biomarkers or a valid genetic model for depression further contributes to the difficult task of studying neuropsychiatric disorders, including PSD, in a preclinical setting [59]. These challenges highlight the need for continued refinement of animal models and methodologies to enhance the translational potential of PSD preclinical research.

The purpose of this review was to conduct a thorough investigation of the current literature on murine models for PSD, highlighting both the strengths and weaknesses of these models, as well as the behavioral assessments employed. Our goal is to identify effective animal models and behavioral evaluations so that their inherent limitations can be overcome. Further, this review aims not only to underline the tools available for such studies but also discuss the challenges faced in translating these preclinical results into the clinic. 

## 2. Materials and Methods

**Figure 1:**
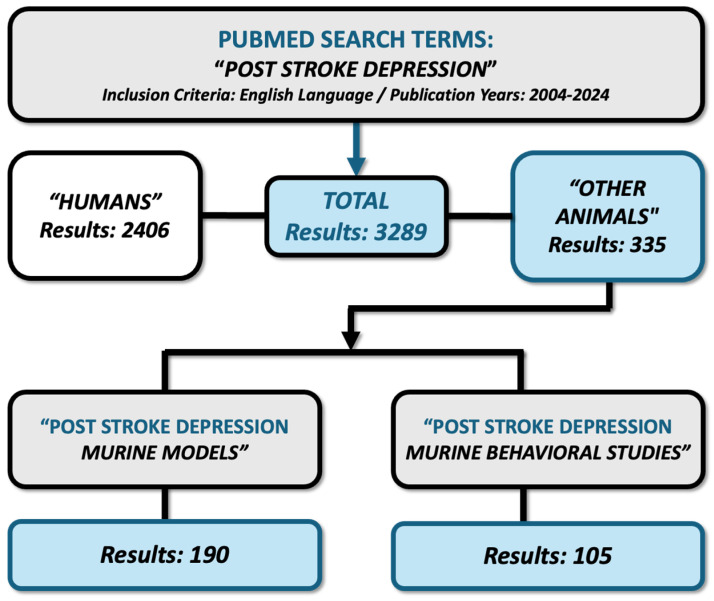
Flowchart of the literature search using PubMed.

We have used PubMed and ClinicalTrials.gov in order to identify a thorough range of relevant scientific manuscripts. Importantly, our search extended to encompass animal models of PSD and various behavioral studies and provides a comprehensive overview of the methods used in exploring PSD. The search terms employed mostly included “post-stroke depression murine models” and “post-stroke depression murine behavioral studies”. Also, to ensure thorough coverage, we used derived terms such as “vascular depression”, “behavioral tests’, “cognition in rodents”, “social activity in rodents”, “motor function in rodents”, “stroke in rodents”, “chronic mild stress”, “depressive-like behavior”, and “anxiety and depression assessments in murine models”. Additionally, we manually reviewed the reference lists of all sourced articles to uncover further citations that the initial database search might have missed. Although additional searches yielded a large number of results, we focused on these specific terms to maintain the relevance and manageability of manuscripts. We specifically chose articles published within the past two decades, spanning from 2004 to 2024, thus ensuring the inclusion of up-to-date research findings. A language criterion was established to only consider articles written in English. This review primarily adopts a narrative approach and does not adhere to the Preferred Reporting Items for Systematic Reviews and Meta-Analysis (PRISMA) guidelines. However, the article selection process was meticulously structured, ensuring that included articles were scrutinized by a panel of three independent reviewers (Figure 1). 

## 3. Incidence and Prevalence of PSD

The incidence and prevalence of PSD exhibit considerable variability in the literature due to differences in methodology, population demographics, and stroke characteristics that underscore its recognition as a frequent consequence of cerebral ischemia [21]. Studies show that between 39% and 52% of stroke survivors experience symptoms of PSD within the first five years after the ischemic event [60]. Studies have also shown that the risk of developing depression is highest within the initial months following stroke [24], with a gradual decline over time [61]. This acute onset suggests a complex interplay of biological, psychological, and social factors, which includes inflammatory mechanisms, the hypothalamic–pituitary–adrenal (HPA) axis, limited capacity for independent living, economic status, negative life events, family burden, and social family support. Collectively, they all contribute to the development of PSD [51]. Additionally, the prevalence of PSD tends to be higher in individuals with more severe strokes, those with a history of depression, and those experiencing greater functional impairment post-stroke [60]. While certain studies suggest that the prevalence of depression after stroke does not significantly differ between sexes, it appears to vary depending on the individual’s pre-stroke depression status [62]. But, other reports state that elderly female stroke patients are 20% more likely to develop PSD in comparison to males [63]. Collectively, all of the aforementioned point to the need for new, effective, and preventive therapeutic approaches [64].

## 4. Risk Factors Involved in PSD

### 4.1. Stroke Characteristics and Lesion Localization

**Figure 2:**
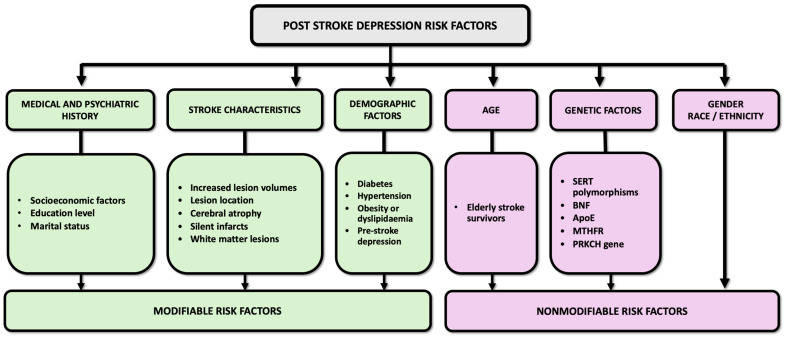
Diagram showing risk factors associated with PSD. Serotonin transporter (SERT), brain-derived neurotrophic factor (BNF), apolipoprotein E (ApoE), methylenetetrahydrofolate reductase (MTHFR), and Protein Kinase C Eta Gene (PRKCH).

Increased lesion volumes, cerebral atrophy, silent infarcts, and white matter lesions are factors that may correlate with an elevated risk of PSD [65] (Figure 2). Lesion location within the brain is a critical determinant of PSD risk, as specific brain regions play distinct roles in mood regulation and emotional processing. Previous studies have demonstrated that the site of stroke lesions (i.e., prefrontal cortex, limbic area, and basal ganglia) significantly influences the likelihood of developing PSD [56,66,67]. The neuroanatomical model proposed by Soares in 1997 also links mood disorders, including PSD, to specific brain regions such as the frontal lobe, basal ganglia, amygdala–hippocampus complex, and thalamus [68]. This model emphasizes the importance of the basal ganglia for the transmission of 5-HT and DA and how ischemic damage to brainstem monoaminergic nuclei or their projections can decrease monoamine levels, affecting mood and cognition. Damage to the left frontal cortex is also often linked to depressive symptoms; individuals with lesions in the left hemisphere may be particularly susceptible to developing depression and anxiety following a stroke [69]. Although there are data suggesting a significant risk for depression following right hemisphere strokes during the subacute phase [70], it appears that a higher risk is associated with left hemisphere lesions [71]. Overall, while the link between the lesion site and PSD is generally acknowledged, the exact relationship remains subject to ongoing research. 

### 4.2. Demographic Factors 

Demographic factors play a significant role in influencing the risk and occurrence of PSD. Gender disparities exist in the prevalence of PSD, with women generally exhibiting higher rates of depression following a stroke compared to men [63,72,73]. Hormonal fluctuations, psychosocial factors, and differences in coping mechanisms may contribute to these gender differences observed [72]. Socioeconomic factors, including education level, income, and access to healthcare resources, also play a crucial role in the development and course of PSD [55]. Stroke survivors from lower socioeconomic backgrounds are more likely to experience financial strain, social isolation, and limited access to mental health services, exacerbating their risk of developing PSD [74]. Marital status is another factor associated with PSD risk, with unmarried individuals, including those who are divorced, widowed, or single, exhibiting higher rates of depression or suicidal ideations following a stroke [75].

### 4.3. Age

Studies have shown that the incidence and severity of PSD increase with advancing age, highlighting the importance of age-related considerations in its management [76,77]. The incidence of PSD is especially high in elderly stroke survivors [78], with 34% in stroke patients vs. 13% in the matched general population [79]. As most candidate therapies for PSD are being developed and studied on young animal models [34], age, which is proven to be detrimental to recovery, may aid in ushering a certain degree of accuracy to existing murine models, thus reducing the translational gap between preclinical and clinical studies [80]. Although a vast array of risk factors such as diabetes [81], hypertension [82], obesity, or dyslipidemia [83] are linked to ischemic brain injury, age represents a non-modifiable risk factor that is not only linked to an increased susceptibility to stroke but also to significantly decreased functional recovery [76,77,84]. It is, therefore, considered that age is a key modulatory factor for both stroke and PSD.

### 4.4. Genetic Factors

Genetic factors can also contribute to the complex interplay of biological and environmental determinants underlying PSD [85]. Polymorphisms within the serotonin transporter gene (SERT) are associated with PSD in stroke survivors [86], and BDNF is a significant contributor to the pathophysiological mechanisms underlying PSD [87]. Research also suggests that both apolipoprotein E (ApoE) and methylenetetrahydrofolate reductase (MTHFR) may contribute to an increased risk of major depressive disorder after a stroke. Interestingly, the catechol-O-methyltransferase (COMT) gene, crucial for DA degradation in the brain, along the 5-HT2A receptor gene that is crucial in serotonin signaling, were studied for their roles in ADHD, schizophrenia, mood regulation, and aggressive behavior [88]. Given their implications in such diverse neurological and behavioral conditions, it is particularly compelling to explore these genes within the context of PSD. The COMT and 5-HT2A genes, through their respective pathways in DA and 5-HT metabolism and signaling, could provide insightful connections to the neuropsychiatric and emotional challenges seen in PSD. While advancements in this area have been limited, certain genes such as protein kinase Cη (PRKCH), angiotensin-converting enzyme, and apolipoprotein may also play an important role in the development of vascular-related depression [89,90]. Additional investigations are ongoing to further delineate the precise genetic factors influencing genetic susceptibility to PSD [91].

### 4.5. Medical and Psychiatric History

The burden of comorbidities and the severity of pre-stroke functional impairment can influence the development and severity of PSD [81,82,83]. Additionally, a history of psychiatric disorders is a strong predictor of PSD [92]. It is concerning that one out of every six stroke patients has experienced PSD [93]. Attention to lifestyle factors, stress management, and social support networks can offer additional layers of prevention and therapeutic intervention, potentially mitigating the impact of pre-existing conditions and enhancing the overall wellbeing of stroke survivors. After-stroke rehabilitation programs, including progressive resistance training [94], modified cardiac rehabilitation [95], vocational rehabilitation [96], family-based programs [97], aquatic [98], music [99], cognitive behavioral therapy [100], repetitive transcranial magnetic stimulation [101], pet therapy [102], and robotic devices [103], can all significantly improve the quality of life and prevent or reduce PSD symptoms. 

## 5. Valid Animal Models for PSD

**Figure 3:**
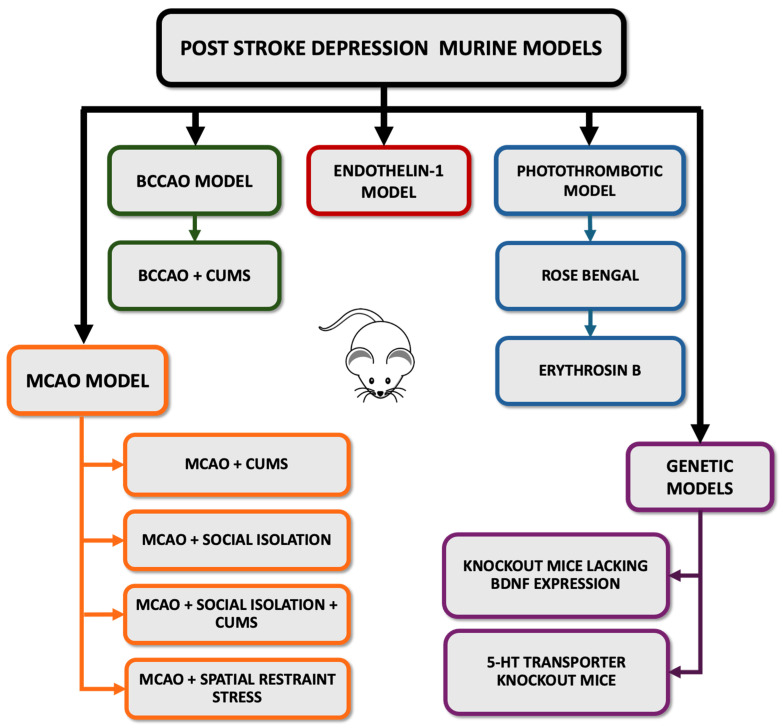
Diagram showing murine models of PSD. Bilateral Common Carotid Artery Occlusion (BCCAO), chronic unpredictable mild stress (CUMS), Middle Cerebral Artery Occlusion (MCAO), 5-Hydroxytryptamine (5-HT, serotonin), and brain-derived neurotrophic factor (BDNF).

PSD poses significant challenges in behavioral assessment due to the subjective nature of its psychological and physiological symptoms [33]. This subjectivity further complicates the development of accurate and reliable experimental animal models capable of capturing the full spectrum of human symptoms. Researchers seek to navigate this complexity by creating models that not only mimic the neuroanatomical and biochemical alterations following a stroke but also elicit behavioral changes consistent with depressive phenotypes. This endeavor extends to identifying and validating behavioral assays that can reliably quantify depressive-like behaviors in animals. However, the translation from animal models to the human condition is fraught with challenges, including differences in brain structure and function, the complexity of human emotions, and the influence of environmental and social factors on mental health [104]. Moreover, the heterogeneity of stroke in terms of location, severity [65], and individual patient factors, like pre-existing mental health conditions [93], further complicates the accuracy of modeling PSD. As such, ongoing research aims to refine these models, increase their translational value, and ultimately, enhance our understanding of PSD pathophysiology. Below, we outline several useful animal models that can contribute to PSD-related research (Figure 3). 

### 5.1. Middle Cerebral Artery Occlusion (MCAO) Model

The MCAO model is one of the most widely used protocols to mimic stroke in rodents [105]. In this model, the middle cerebral artery is occluded either transiently or permanently [106], resulting in focal cerebral ischemia and subsequent stroke-like symptoms, including depressive-like behavior [107]. This model allows for precise quantitation of neurological deficits, infarct sizes, and the impact of therapeutic interventions. However, the MCAO model comes with its own set of challenges, one of them being the technical complexity of the procedure, which demands high surgical expertise to ensure consistency in ischemia severity and reduce variability among animals [108]. The procedure entails slowly lifting the right MCAO using a tungsten hook connected to a micromanipulator and then thermocoagulating it. Both common carotid arteries are subsequently ligated for 90 min. After this period, the common carotid arteries are reopened [109,110]. The muscle and soft tissue are then repositioned, and the skin is sutured. Initially, the occlusion is confirmed visually, followed by measuring and comparing the blood flow to normal levels. An 80% reduction in blood flow is deemed successful [111]. Researchers use various durations of MCAO to mimic different levels of stroke severity [112,113]. This approach allows them to investigate the underlying mechanisms of brain injury and assess potential therapeutic interventions. The occlusion periods can range from 20 [114], 30 [115], 45 [116], 50, 60, 70 [113], 90 [117], and 120 [118] minutes to permanent occlusion [119]. However, the success rate of PSD modeling using the MCAO model alone is limited. While some mice/rats with MCAO may exhibit depressive behaviors, these are often short lived and can include anxiety-like behaviors. To achieve more consistent and prolonged depressive behaviors, it is necessary to combine MCAO with other techniques, such as chronic unpredictable mild stress (CUMS). This combined approach enhances the validity and reliability of PSD models by more accurately replicating the complex pathophysiological and behavioral aspects of PSD. Such methodologies help to create a more comprehensive model, allowing for more in-depth studies and better understanding of PSD mechanisms [35].

### 5.2. MCAO Model Combined with CUMS

Animals undergoing MCAO followed by chronic mild stress exhibit a heightened severity of depression-like behavior compared to those undergoing MCAO alone [35]. This model is advantageous as it mimics the chronic stress often experienced by stroke survivors and allows for the study of PSD. This model was successfully demonstrated in both rats [120,121] and mice [122,123]. Stressors may include mild physical stress (electric shock, tail clamp, restraint stress, forced swim), social stress (isolation, overcrowding), and environmental stress (altered light–dark cycle, food or water deprivation, cage tilt or cage shaking, wet bedding). The stressors and duration for implementing CUMS can vary significantly, between 21 [124], 28 [125,126,127], 35 [128,129,130], 42 [131,132], 49 [133], and 56 days [134,135], involving both group and individual housing (Table 1). 

### 5.3. MCAO Model Combined with Social Isolation

Rodents are highly social mammals, and single housing, mimicking social isolation, can lead to various behavioral and physiological changes, including depressive-like behaviors [136]. Mice that underwent individual housing for 14 days following a stroke exhibited exacerbated depressive-like behavior compared to pair-housed mice [137].

### 5.4. MCAO + Social Isolation + CUMS Model

The MCAO + social isolation + CUMS protocol stands out as the most extensively utilized approach for modeling PSD in preclinical studies [38]. Following surgery, the animal is individually housed to facilitate postoperative recovery and, subsequently, it undergoes exposure to various stressors throughout the course of the CUMS procedure [138,139,140,141]. Previously, it was shown that even without a stroke procedure, social isolation is considered the most fitting housing condition during the CUMS regimen for studying depression [127,142]. This integrative model effectively mimics the human experience of PSD, incorporating both the physical impact of a vascular event and the psychological stress from environmental changes, thereby providing a comprehensive framework for exploring the complex interplay between physical and mental health and environmental factors in post-stroke outcomes.

### 5.5. MCAO Model Combined with Spatial Restraint Stress

Restraint stress has been demonstrated to impair sociability in rodents [143] and has also been successfully used as a stressor during the CUMS procedure for 2 h [126], 4 h [131], or 6 h [129]. Following MCAO, restraint stress-induced depressive-like behavior, as assessed through behavioral tests, was applied, which resulted in dull hair color and a poor general state [144].

### 5.6. Bilateral Common Carotid Artery Occlusion (BCCAO) Model

The BCCAO model, introduced as a stroke model, entails ischemic white matter and eye injury and is simpler to establish compared to MCAO [145]. Following BCCAO induction, depressive-like behavior was observed in 5-week-old Balb/c mice [146,147,148], albino mice [149], and Wistar rats [150] without the need for an additional method to induce PSD. 

### 5.7. BCCAO Model Combined with CUMS

Recent research efforts have employed the BCCAO model, alongside a two-week CUMS protocol, in order to develop a murine (C57B16J) model of PSD [151]. While this model represents a significant advancement in replicating the complexities of PSD, it is important to acknowledge the inherent variability in response among different rodent strains and species.

### 5.8. Intracerebral Injection of Endothelin-1 (ET-1)

ET-1, a powerful vasoconstrictor produced internally during ischemic stroke, plays a crucial role in neuronal damage and subsequent disability [152]. The administration of ET-1 into the left medial prefrontal cortex (mPFC) of mice has been shown to cause a pronounced and lasting anxiety and depressive phenotype, establishing its potential as a murine model for PSD [153]. In contrast, experiments conducted in rats have demonstrated that ET-1 leads to anxiety-like behaviors but not depressive-like behaviors. This disparity suggests that additional damage to a secondary brain area might be necessary to elicit a depression phenotype in rats, highlighting the complications of modeling PSD across different rodent species [154].

### 5.9. Photothrombotic Model

Photothrombosis involves the induction of focal cerebral ischemia by illuminating a photosensitive dye in the presence of a light source, leading to thrombus formation and vascular occlusion [155]. One commonly used dye is Rose Bengal, which, when activated by green light (560 nm) [156], generates reactive oxygen species, leading to clot formation and vascular occlusion [157]. Another example is Erythrosin B, which operates in a similar manner but is activated by a different wavelength (near 660 nm) [158] and offers flexibility in experimental setups. These dyes are selected for their high quantum yield of singlet oxygen production, a key factor in inducing rapid and targeted vascular occlusion [159]. The photothrombosis model provides exceptional precision in dictating both the location and extent of ischemic lesions, a feature that has been instrumental in linking specific brain areas to behavioral outcomes. This level of control has facilitated research demonstrating that rodents subjected to ischemic lesions exhibit behavioral changes reminiscent of depression [38].

### 5.10. Genetic Models of PSD

Genetically modified animals with alterations in specific genes implicated in depression or stroke pathophysiology can also be valuable for studying PSD. For example, knockout mice lacking BDNF [160] and the 5-HT transporter [161] exhibit depressive-like behaviors and impaired neurogenesis. However, future research should aim to elucidate the complex interactions between genetic factors, environmental stressors, and stroke-induced neurobiological changes to enhance the understanding of PSD in these knockout murine models.

## 6. Assessing Symptoms and Behaviors: Key Tests for Studying PSD 

**Figure 4:**
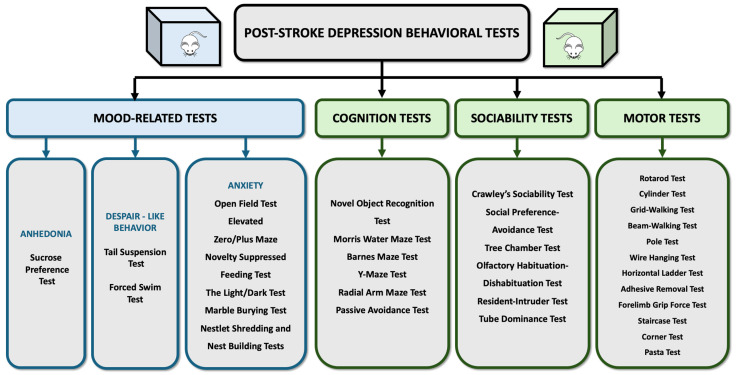
Behavioral assessment methods for post-stroke depression in rodent models.

This section outlines a range of behavioral tests crucial for investigating the pathophysiological impacts of stroke and for evaluating the accuracy of animal models and the efficacy of therapeutic interventions (Figure 4). These tests encompass motor function evaluations, assessing coordination and muscle strength, as well as cognition, which measures memory and learning capabilities. Additionally, social behavior tests are discussed, which are used to examine interaction patterns and anxiety levels among animals.

### 6.1. Anhedonia Test: Sucrose Preference Test (SPT)

Anhedonia, defined as a diminished capacity to experience pleasure or interest in previously rewarding activities, is a hallmark symptom of depression that can significantly impact prognosis and complicate patient recovery [162]. In rodent models, the assessment of anhedonia primarily relies on measuring the preference for sucrose, a simple yet effective indicator of pleasure-seeking behavior. SPT involves providing each mouse with two identical bottles: one containing a sucrose solution and the other water. The animals are then allowed to choose freely between them [163]. A marked decrease in sucrose consumption is interpreted as an expression of anhedonia [164,165]. The test procedure encompasses several variables, including housing during the habituation, concentration of sucrose solution, period at which the experiments are performed (light/dark), type of habituation to the SPT procedure, type of food/water deprivation during the test, and test duration [166]. SPT has proven to have satisfactory results in various studies on different animal models of PSD. For example, it revealed severe anhedonia after MCAO and 18 consecutive days of CUMS [123]. Also, MCAO and spatial restraint stress led to a decreased percentage of sucrose consumption compared to stroke alone [113]. Lastly, MCAO followed by social isolation showed a significant increase in sucrose consumption for post-stroke pair-housed mice compared to those that were socially isolated [137].

### 6.2. Depression-like Behavior Tests: Forced Swim Test and Tail Suspension Test

Depression encompasses a complex array of symptoms, including profound feelings of hopelessness, persistent sadness, and thoughts of death or suicide [167]. The forced swim and tail suspension tests have been employed as a means to gauge aspects of despair and motivational withdrawal in rodents, which are considered analogs to the human experience of hopelessness and passive resignation.

The Forced Swim Test (FST) is widely utilized for evaluating despair-like behavior in animal models, quantifying the duration of immobility, except for the minimal movements necessary to maintain the animal’s head above water. This behavior is interpreted as a sign of behavioral despair, mirroring aspects of depression [168]. Studies have shown that both MCAO and chronic mild stress can induce despair-like behavior in C57BL/6 mice [35], as well as in albino mice subjected to BCCAO [149].

The Tail Suspension Test (TST) is also used, but only in mouse models. In this test, animals are suspended by their tails, and the duration of immobility is recorded [169], with longer immobility times indicating a higher level of despair. The TST has demonstrated a significant increase in immobility time in C57BL/6 mice five days post-BCCAO [170], NMRI mice 72 h after permanent double ligation of the right common carotid artery [171], and ICR mice following MCAO and spatial restraint stress [172]. 

### 6.3. Anxiety Tests: Open Field/Elevated Zero Maze/Novelty Suppressed Feeding/The Light/Dark/Marble Burying

Before conclusively identifying PSD in animal models, it is critical to evaluate the presence of anxiety, as it often coexists with depression and can influence the overall behavior and response of the animal. Anxiety assessment in rodents can be integrated with social interaction tests or conducted through specific behavioral assays designed to measure anxiety levels, such as the ones outlined below.

The Elevated Zero Maze (EZM) [173,174] and Elevated Plus Maze (EPM) Tests [175] use an elevated apparatus designed to invoke anxiety-related behaviors by exploiting the rodent’s aversion to open and elevated spaces. The EZM, a circular platform divided into open and closed sections, allows for the assessment of anxiety based on the animal’s preference for the safer, enclosed areas over the exposed ones [173,174]. The EPM similarly measures anxiety by recording the time spent in the open arms of a plus-shaped apparatus, with decreased time indicating higher anxiety levels [175]. In experiments involving C57BL/6 mice, the administration of ET-1, a procedure used to mimic stroke conditions, was followed by an assessment using the EPM and indicated that post-ET-1 injection, mice exhibited a marked reduction in the time spent in the open arms of the EPM, suggesting heightened anxiety levels [153]. 

The Open Field Test (OFT) serves as a critical tool for assessing anxiety-like behavior in rodents by tracking the amount of time they spend in the center of an open arena [176]. Anxiety levels in this context are analyzed from the animal’s exploration patterns, with a preference for the periphery over the center indicating higher anxiety [176]. Experiments using C57Bl/6 mice subjected to MCAO and 17 days of chronic mild stress revealed significant anxiety with OFT, as demonstrated by reduced central area exploration [35]. Similarly, MCAO followed by periods of social isolation also led to a noticeable decrease in the time these mice spent in the center [137].

The Novelty Suppressed Feeding Test (NSFT) is a behavioral assay designed to evaluate anxiety and depression-related behaviors by measuring both the amount of food intake and the delay before the animal engages with a new, highly palatable food item [177]. This test is predicated on the natural conflict between the fear of a novel environment and the motivation to eat, with increased latency and reduced food consumption indicating heightened anxiety or depressive states [177]. Studies involving C57BL/6 mice demonstrated that after microinjection of ET-1, mice exhibited a decreased interest in food and a significant delay before beginning to eat the novel food, suggesting an increase in anxiety or depressive-like behavior [153]. Similarly, following an MCAO procedure, mice showed increased latency to approaching and consuming the food pellet three weeks post-ischemia [178].

The Light/Dark Test (L/D Test) measures an animal’s willingness to explore or avoid new environments [179,180]. This test utilizes a chamber divided into illuminated and dark sections and allows measurement of the time taken to enter the light compartment and the number of transitions between compartments, reflecting the animal’s exploratory behavior and its aversion to brightly lit areas, respectively [179]. A preference for spending more time in the dark compartment is interpreted as an indication of anxiety. The L/D Test has been effectively employed in studying PSD in murine models [153].

In the Marble Burying Test, animals are placed into a cage layered with bedding, in which marbles or similar small objects are evenly distributed. Researchers then measure the number of marbles the rodent buries within a specified period. A tendency to bury more marbles is interpreted as an indication of heightened anxiety or compulsive tendencies, providing a straightforward method for assessing these behaviors [181,182].

Nestlet Shredding and Nest Building Tests also serve as valuable tools for determining stress levels in rodents [183]. These tests examine the natural nesting behavior, where rodents are provided with materials, like cotton nestlets, to build nests. The extent and quality of the nest constructed, along with the degree of shredding of the provided materials, are indicative of the animal’s well-being, with poor nesting behavior suggesting elevated stress or discomfort [183].

### 6.4. Social Withdrawal Tests: Crawley’s Sociability/Social Preference-Avoidance/Tree Chamber/Olfactory Habituation-Dishabituation/Resident-Intruder/Tube Dominance 

Social withdrawal is a critical symptom observed in numerous psychiatric disorders, notably depression [184]. The array of behavioral tests deployed to study this condition in rodents not only sheds light on the underlying mechanisms but also holds significant value in assessing social withdrawal symptoms associated with PSD [185,186,187]. These tests, designed to evaluate interactions among rodents or their response to social stimuli, provide insight into changes in social behavior potentially indicative of PSD and are briefly described below. 

Crawley’s Sociability Test is a key method for evaluating social behavior and novelty preference in rodents [188]. This procedure involves an initial interaction phase where the test animal is given the opportunity to interact with a “stranger” mouse that it has not previously encountered. After a 10 min interaction period, a second, novel “stranger” mouse is introduced into the apparatus. The subject mouse is then observed to see whether it shows a preference for the already-investigated unfamiliar mouse or the new, novel unfamiliar mouse. This test provides valuable insights into the nuances of rodent social behavior, particularly after experiencing a stroke.

The Social Preference–Avoidance Test is used for both mice and rats and is designed to measure the dynamics of social interaction, specifically the speed of approach or avoidance displayed by the animal during the test [189,190]. This test provides insight into the social tendencies of rodents, offering a nuanced view of how they navigate social spaces and whether they show a propensity towards engaging with or avoiding other animals.

The Three Chamber Test is employed to assess sociability and social memory by evaluating a rodent’s preference for an unfamiliar conspecific or an inanimate object and its preference for a new or a familiar conspecific [191]. This test effectively distinguishes between the animal’s interest in social interactions and its ability to recognize and differentiate between familiar and unfamiliar individuals. 

The Olfactory Habituation–Dishabituation Test, while initially utilized in evaluating autistic behaviors in mice, serves a broader purpose in assessing the olfactory system, which is vital for studying sensory processing in the brain [192]. Mice naturally exhibit a preference for novel scents over familiar ones [193]. Assessment of the olfactory system has proven useful for studying sensory processing in the brain [194] but also serves as a valuable tool for assessing social interaction, memory, and anxiety [195]. Social interaction is conditioned by the level of anxiety, while anxious behavior is often associated with depression [196].

The Resident–Intruder Test is another significant behavioral assay where a resident rodent is confronted with an unfamiliar “intruder” in its environment. The resultant behaviors, ranging from aggressive to affiliative, are observed and scored. This test is instrumental in evaluating behaviors such as territorial aggression, social dominance, and social recognition memory [197].

The Tube Dominance Test is primarily employed to measure social hierarchy and dominance in mice [198]. After a day of habituation and training, two mice enter a narrow tube from opposite sides and meet in the middle. The mouse that persuades the other to retreat is deemed the winner, respectively dominant. The test was successfully used in a mouse model of depression involving CUMS [199] and could provide additional insights regarding PSD.

### 6.5. Cognitive Impairments Tests: Morris Water Maze/Barnes Maze/Y-Maze/Novel Object Recognition/Radial Arm Maze/Passive Avoidance

When dealing with depression, individuals often experience cognitive impairments, including difficulties with memory and attention, loss of concentration, and problems with learning processes [200]. These cognitive symptoms are critical components of the overall clinical representation and can significantly affect the quality of life and daily functioning. Recognizing the importance of these symptoms, cognitive assessment in PSD has also been approached through various behavioral tests in research settings that are described below. 

The Morris Water Maze Test is a widely recognized method for evaluating spatial learning and memory in rodents. Animals are placed in a sizable water pool with a platform submerged beneath the surface. Rodents must navigate using spatial cues in order to find the platform. Through repeated trials, they gradually learn and remember the platform’s location, demonstrating spatial memory retention [201]. Pre-experimental learning trials are often conducted to familiarize rodents with the task [202,203]. Studies have shown an increased latency to find the platform in MCAO mice in the 3rd [178] and 6th week after stroke [35].

The Barnes Maze Test offers an alternative to the Morris Water Maze, utilizing a dry, less stressful environment for the rodent. This test involves a circular platform with multiple holes around its edge, one of which leads to an escape box. Rodents are required to navigate using spatial cues to find this escape route, providing insights into their spatial learning and memory capabilities [204].

The Y-Maze Test is another critical tool that measures the willingness of rodents to explore a new environment by recording the number of arm entries and the sequence of these entries to assess spontaneous alternation behavior [205]. This test is particularly useful for observing short-term memory by analyzing the percentage of correct alternations made by the rodent, reflecting its ability to remember previously visited arms [205]. Research involving C57BL/6 mice post-MCAO surgery and subsequent individual housing revealed a decrease in the percentage of correct alternations in the Y-maze, suggesting impairments in spatial working memory as compared to pair-housed mice [137]. 

The Novel Object Recognition Test (NORT) serves as a behavioral assay for evaluating memory capability, particularly recognition memory [206]. Initially, animals are allowed to familiarize themselves with an arena containing two identical objects [207]. Subsequently, one of the original objects is replaced with a novel object, and the animal’s interaction with both objects is observed [208]. A preference for exploring the novel object over the familiar one is typically indicative of healthy recognition memory, as it suggests the animal remembers the original object and finds the new one more interesting [207,208]. Studies employing NORT have demonstrated its utility in detecting memory impairment. For example, socially isolated mice subjected to MCAO showed impairment in recognition memory, as evidenced by their equal interest in exploring both novel and familiar objects. This lack of preference for the novel object indicates a difficulty in recognizing or remembering the previously encountered object, underscoring the impact of social isolation and stroke on cognitive functions [137]. 

The Radial Arm Maze Test typically consists of a central platform with multiple arms extending outward, resembling the spokes of a wheel [209]. At the end of each arm, food rewards or other incentives are placed to motivate the rodent. The animal is placed in the central area and must efficiently navigate through the maze to collect the rewards. Successful navigation involves remembering which arms have already been visited to avoid unnecessary revisits, thereby demonstrating the animal’s ability to learn and remember spatial information [210].

The Passive Avoidance Test is a specific behavioral assay used to assess learning and memory after stroke [211]. Rodents are typically placed into a two-compartment apparatus, one illuminated and one darkened. Initially, the animal is allowed to explore both compartments freely. After a predetermined period of time, usually during the training phase, the animal receives a mild aversive stimulus (i.e., foot shock) upon entering one of the compartments, typically the darkened compartment. This creates an association between the aversive stimulus and the compartment. During the testing phase, the animal is again placed in the apparatus and allowed to freely explore both compartments. The latency to enter the aversive compartment is recorded. Animals with intact memory will exhibit a longer latency to enter the aversive compartment due to their association with the stimulus [212].

### 6.6. Motor Function Tests: Rotarod/Cylinder/Grid-Walking/Beam-Walking/Pole/Wire Hanging/Horizontal Ladder/Adhesive Removal/Forelimb Grip/Staircase/Corner/Pasta

Assessing motor function in murine models of PSD is essential for comprehending the effects of stroke. Accordingly, a range of tests has been introduced to measure various aspects of motor skills, including coordination, balance, skilled locomotion, muscle strength, and forelimb functionality, and these are described below and shown in Figure 4.

The Rotarod Test stands as the benchmark for evaluating motor function, particularly coordination and balance in mice [43,213]. In this test, mice are placed on a rod that rotates at a controlled speed. The duration for which each mouse remains on the rod before falling is recorded, serving as a measure of its motor coordination and balance [214]. Additionally, a variation of the Rotarod, the RotaWheel, has emerged as a novel experimental tool for assessing locomotion in mice [215,216]. This apparatus offers a new dimension to the evaluation of motor skills, providing insights into locomotion abilities as well as endurance [215,216].

The Cylinder Test is a crucial assessment for evaluating forelimb asymmetry, particularly in the context of sensorimotor function following stroke [217]. In this test, mice are placed inside a transparent cylinder, and the use of their forelimbs during vertical exploration or rearing movements is carefully observed and recorded. Stroke-induced deficits can lead to a noticeable asymmetry in forelimb use, where the animal might predominantly use one limb over the other, reflecting the impairment of sensorimotor function to one side of the body. This test has frequently been adopted to assess motor function recovery or decline after stroke, providing valuable insights into the extent of motor rehabilitation or the effectiveness of therapeutic interventions aimed at mitigating motor function deficits [153]. The ability of this test to detect subtle changes in limb usage offers a sensitive measure of motor skills and recovery.

The Grid-Walking Test is specifically designed to evaluate skilled locomotion and motor coordination [217]. Mice are placed on a grid that features widely spaced holes. As the animals navigate across the grid, the incidence of foot slips through the holes is recorded [218]. This approach allows for precise quantification of motor deficits, particularly those affecting coordination and the ability to perform complex movements. Stroke-induced impairments are often manifested as an increase in the number of foot slips, indicating a loss of motor control or diminished spatial awareness [217,219].

The Beam-Walking Test assesses balance, coordination, and skilled locomotion [220], and it requires a raised-beam apparatus and training sessions for the subjects [221]. Animals have to traverse a narrow beam to reach a secure platform, with their performance providing insight into their motor capabilities. Both the time taken to cross the beam and the incidence of foot slips during the attempt are key metrics for assessing the presence and extent of motor deficits, particularly those resulting from stroke-induced damage. When the Beam-Walking Test was been applied to Sprague Dawley rats following MCAO, significant functional impairments were documented [222,223]. 

The Pole Test involves placing animals at the top of a vertical pole, where they are trained to perform a turnaround maneuver before descending the pole headfirst. Evaluation focuses on the time it takes for the animal to initiate and complete the turnaround maneuver, as well as the descent [43]. This approach allows researchers to assess motor coordination, agility, and the animal’s overall ability to control and execute complex motor tasks. The test proved useful in the evaluation of mice after MCAO [224].

The Wire Hanging Test examines the forelimb motor strength of mice after stroke [224]. The mice undergo training to hang their bodies from a steel wire, which measures 2 mm in diameter, solely using their forelimbs. This training spans two days, including three trials per day. The average holding time across the three trials is calculated and analyzed [225]. A lower holding time is indicative of a decrease in motor strength.

The Horizontal Ladder Test is used to evaluate walking ability [226]. The animals are trained to cross the ladder from a neutral cage to reach their home cage [227]. During the test, the number of successful steps, slips, or missed steps is measured [228]. These assessments provide valuable insights into the animals’ motor skills and coordination abilities.

The Adhesive Removal Test is another method for evaluating sensorimotor deficits and somatosensory function in rodent models after stroke [229,230]. Small adhesive stimuli, such as sticky tape or adhesive-backed dots, are placed on the forepaws of the animal, and their ability to detect, remove, and discriminate between the stimuli is assessed [231].

The Forelimb Grip Force Test is used to measure muscle strength, providing a quantitative assessment [223]. Studies have reported a decrease in grip force for both the right and left hind paws in C57BL mice 7 days following Distal Middle Cerebral Artery (DMCA) occlusion. Interestingly, this reduction in grip strength was not observed 28 days post-stroke, indicating some degree of recovery over time [232]. Additionally, rats subjected to MCAO combined with CUMS exhibited a significant decrease in grip force 22 days after the injury [223].

The Staircase Test is commonly used with rodents to assess skilled reaching and grasping abilities, particularly in the context of examining motor function and recovery after stroke. In this test, rodents are typically placed in a cage equipped with a staircase apparatus consisting of a series of steps with food rewards placed on each step. The animals’ ability to navigate the staircase and retrieve the food rewards offers insight into their skilled reaching and grasping capabilities and overall motor recovery [233].

The Corner Test also provides valuable insights into motor asymmetry and sensorimotor function [43]. Following a stroke, the rodent is placed near a corner of a testing apparatus, typically a rectangular or triangular enclosure with two converging walls forming the corner. As the rodent approaches the corner, it tends to turn in the direction of its more impaired side, leading to a higher frequency of turns toward the affected side compared to the unaffected one. Researchers observe and record the direction of the turns as well as any asymmetry in movement patterns [234]. This method allows for the assessment of both the preference in turning direction and any motor deficits that might influence this preference, offering a direct indication of unilateral sensorimotor impairment.

The Pasta Test provides valuable insights into the motor abilities and functional recovery of rodents following neurological insults [235]. Animals are typically given a piece of pasta to manipulate and eat. The researchers then observe and analyze the rodent’s behavior, focusing on the symmetry and effectiveness of its forepaw movements during the manipulation and consumption of the pasta. Impairments in fine motor skills or asymmetrical use of the forepaws can indicate deficits in manual dexterity, indicative of neurological damage or dysfunction [236].

Motor dysfunction and depressive symptoms frequently coexist in patients following a stroke, and there is evidence to suggest that these motor deficits can influence the onset and severity of depressive symptoms [237]. The DigiGait system provides detailed and quantitative assessments of gait and offers a unique opportunity to study these motor abnormalities in stroke models [43]. Although DigiGait has not been extensively used specifically for PSD research, its ability to precisely measure changes in gait and coordination could be invaluable in understanding the relationship between motor deficits and depressive behaviors post-stroke [238]. Incorporating DigiGait assessments in PSD studies could enhance our understanding of how motor impairments contribute to or exacerbate depressive symptoms. Future research may explore this intersection, potentially leading to more comprehensive therapeutic strategies that address both the motor and psychological aspects of post-stroke recovery.

## 7. Pathophysiological Mechanisms Involved in PSD

The pathophysiological mechanisms underlying PSD are multifactorial, involving biological, neurochemical, and psychosocial factors that interplay to reveal depressive symptoms following a cerebrovascular event. Stroke lesions in critical brain areas, notably the prefrontal cortex, limbic system, and basal ganglia, play a pivotal role in disrupting neurotransmission pathways essential for mood regulation, thus contributing to the onset of PSD [56]. Specifically, lesions in the left hemisphere (left frontal cortex and basal ganglia) are correlated with a higher incidence of depression, which is attributed to diminished levels of 5-HT and norepinephrine (NE) [56]. In contrast, another study also points to the right hemisphere’s involvement in PSD, particularly during the subacute phase (1–6 months) [70]. This neurotransmitter hypothesis further underscores the role of monoamines (NE, 5-HT, DA) in mood regulation, as ischemic injury notably decreases their production and availability. Ischemic lesions can disrupt the axons containing biogenic amines that ascend from the brainstem to the cerebral cortex, resulting in reduced levels of monoamines in limbic structures found in the frontal and temporal lobes, as well as the basal ganglia. In turn, this influences motivation-related behaviors such as salience detection, reward and punishment learning, processing incentives, decision making, goal-directed actions, and regulation of anxiety levels [239]. Additionally, genetic predispositions, such as the 5-HTTLPR genotype, also modulate susceptibility to PSD [240,241,242]. 

Inflammatory cytokines also play significant roles in the pathophysiology of both stroke and depression. Specifically, the IL-10 -1082A/A genotype has been linked to PSD in general, while the IL-4 + 33C/C genotype has shown an association with major PSD [243]. These genetic variants highlight the interplay between immune response and psychiatric outcomes following stroke, suggesting potential genetic markers for susceptibility to PSD subtypes. Inflammation is a critical contributor, with elevated levels of pro-inflammatory cytokines (IL-1β, IL-4, IL-8, TNF-α) [244,245] and the activation of pathways, such as NLRP3, signifying an influence on PSD pathophysiology [246]. Neuroendocrine dysregulation, especially concerning the HPA axis and resultant elevated cortisol levels, also contributes significantly to PSD, highlighting the neuroendocrine system’s critical role in mood regulation [247,248]. Poor post-stroke prognosis is linked to alterations in the HPA axis, elevated levels of catecholamines and natriuretic peptides, and reduced levels of melatonin and IGF-1 [249]. Moreover, neurotrophic factors, like brain-derived neurotrophic factor (BDNF) and glial cell line-derived neurotrophic factor (GDNF), essential for neuronal health and regeneration post-injury, are also linked to PSD development, with variations in their levels and methylation status closely associated with depressive outcomes post-stroke [250,251]. Together, these mechanisms offer a comprehensive insight into the intricate number of factors contributing to PSD [56]. 

## 8. Strain Differences in Rodents

Strain differences in rodents can significantly influence the manifestation and understanding of PSD [252]. The injection of ET-1 produced a pronounced and persistent anxiety and depression phenotype in C57/BL6 mice [153]. However, in Sprague Dawley rats, it resulted in anxiety-like behavior while it failed to induce depressive-like responses [154]. Even in various rat strains, distinct behavior patterns emerge following stroke. Lewis rats exhibited behavior indicative of depression but not fatigue, whereas Wistar and Sprague Dawley rats displayed behavior indicative of fatigue but not depression [252]. A future and comprehensive analysis of rodent strain-related differences should provide insight into symptom pathophysiology as well as guide researchers in choosing the appropriate mouse or rat strain. Additionally, the development of transgenic animals may also play a critical role in enhancing the translatability of preclinical tests for PSD. Introducing specific genetic modifications will enable researchers to generate animal models that more closely mimic the genetic and molecular aspects of human PSD.

## 9. Translatability of PSD Research

Examining clinical trial registries is essential in order to gain a broad understanding of the progress made in ongoing PSD research. An analysis of PSD research indicates a significant disparity between the abundance of preclinical studies and the relatively limited number of clinical trials. A PubMed search with the keyword “post stroke depression” over the past twenty years produces a total of 3273 entries. This includes 127 meta-analyses, 463 reviews, 180 systematic reviews, and 331 manuscripts related to clinical trials specifically focusing on PSD. 

Further examination of the National Institutes of Health’s (NIH) ClinicalTrials.gov registry (accessed up to and including 20 April 2024] revealed 68 clinical studies at various stages. This search encompassed studies marked both as completed and actively enrolling. Of these, only thirteen are actively recruiting patients, an additional three are not yet enrolling, and one is active but not recruiting. These trials, which primarily involve adult participants of both sexes, range from early Phase 1 to Phase 4, with 43 categorized as “Not applicable” regarding their phase. Fifty-three of these trials are interventional, with a focus on directly modifying participant treatment or behavior to assess efficacy and safety outcomes. There are also 15 observational studies, which typically gather data on PSD without altering the treatment regimen. Additionally, there are four patient registries that systematically collect information about patients with PSD to facilitate future research. None of the studies are classified under expanded access, intermediate-size populations, or treatment IND/Protocol categories, indicating a focus on controlled research settings rather than broad or emergency-use interventions. Remarkably, only four of these studies have reported results on ClinicalTrials.gov. This stark contrast to the abundance of preclinical studies highlights a significant translational gap. As such, there is a pressing need for innovative methodologies and testing that can bridge this divide, which are essential in accelerating the development of effective clinical PSD treatments.

## 10. Future Directions

PSD represents a prevalent and severe human neuropsychiatric complication that impacts a significant number of stroke survivors, presenting challenges not only for the patients but also for the healthcare system and support networks [60]. In this comprehensive review, we compiled and analyzed the current scientific literature on PSD, covering its epidemiological landscape, identifiable risk factors, and the biological and neurological underpinnings. We also explored the tools available for studying PSD in preclinical settings, including various animal (rodent) models and behavioral analyses. Our findings highlighted the complex nature of PSD and underscored the challenges associated with accurately modeling and evaluating its manifestations in preclinical settings. Recent studies have highlighted several pharmacological treatments for PSD. For example, selective serotonin reuptake inhibitors (SSRIs) are commonly prescribed and have demonstrated efficacy in alleviating depressive symptoms in stroke patients. Other antidepressants, such as trazodone and tricyclic antidepressants, like nortriptyline, have also been used with varying degrees of success [253,254]. These treatments work by modulating neurotransmitter levels, thereby reducing depressive symptoms and enhancing the overall quality of life. However, the selection of medication must be carefully considered due to the potential side effects and interactions with other drugs frequently prescribed to stroke patients. Neuromodulation techniques, such as repetitive transcranial magnetic stimulation and transcranial direct current stimulation, along with innovative psychosocial interventions, hold promise as effective treatments and warrant further investigations [254]. Furthermore, effective management of PSD necessitates a multidisciplinary approach that integrates pharmacological treatments with psychological, rehabilitative, and social interventions. This strategy ensures that all aspects of the patient’s condition are addressed, promoting better overall outcomes. Concurrently, ongoing research is focused on developing novel therapeutic strategies, aiming to enhance the efficacy of treatments and minimize side effects [255].

The combination of MCAO with social isolation and CUMS has emerged as a prevalent murine model for studying PSD [38]. The duration of the CUMS procedure can vary for 3, 4, or 6 weeks, reflecting the different intensities and duration of stress exposure in studies that induce depressive-like behaviors [138,139,140,256,257]. Numerous studies utilizing the MCAO+CUMS model explored different aspects of PSD [120,121,122,123], indicating that social isolation on its own can significantly contribute to the induction of rodent depressive-like behavior [136]. This underscores the importance of social factors in the development of depressive symptoms post-stroke, suggesting that the most effective animal models of PSD must incorporate a multifaceted approach, mirroring the complex interplay of human symptoms. Animal models of PSD exhibit distinct pathophysiological changes, which are important for understanding the mechanisms underlying this condition. The MCAO model shows microglial activation [258,259] and elevated levels of pro-inflammatory cytokines in the brain [260,261] that are also involved in the neurodegenerative process [262]. The additional chronic restraint stress and foot shock stress have been observed to decrease BDNF levels [263]. The CUMS model induces the activation of the HPA axis [264], leading to elevated cortisol levels [134]. Finally, the combined models of PSD provide insights into the interaction between ischemia-induced brain damage and stress-induced neuroinflammation, offering a more complex understanding of PSD pathophysiology. By integrating findings from different models, researchers can better elucidate the complex biological processes contributing to PSD and develop more effective therapeutic strategies.

In order to develop a comprehensive animal model of PSD, it is imperative to thoroughly assess all depression-related aspects, such as the association between left hemisphere stroke lesions and the manifestation of depressive symptoms [56,265]. Approximately 40% of individuals who experience left hemispheric infarctions develop depression, typically exhibiting mild to moderate symptoms shortly after the stroke or after several months [266]. However, the hypothesis that the location of the brain lesion influences the risk of PSD is contested, with several studies challenging the notion that depression is more commonly associated with left-hemisphere strokes than with right-hemisphere strokes [70,267,268]. 

Age and gender also represent crucial factors that significantly impact the study of neurodegenerative diseases, including PSD. Research predominantly utilizes young animal models, which may not accurately reproduce the age-related complexities of PSD in humans [34,146,147,148]. Including older animals in these studies will enhance model validity, improve our understanding of age as a critical factor in disease progression and recovery [80], and hopefully reduce the translational gap in clinical applications. Moreover, the prevalence of studies focusing on male rodents [35,113,123,137] may overlook key sex-based dimorphic differences that could influence both the presentation and progression of PSD. Males and females may respond differently to stroke, with potential variations in motor function, mood, cognitive abilities, and memory tasks [269]. Notably, female rodents are more prone to weight loss during chronic social stress and may exhibit heightened anxious behaviors [270]. These differences are modulated by sex hormones, like estrogen and testosterone, which are also known to affect stroke responses and depressive behaviors [271]. For example, estrogen was shown to alleviate depressive symptoms post-stroke [272]; however, recent findings suggest that the estrous cycle in females does not significantly impact behavior or neurogenesis under basal conditions [273,274], indicating that sex differences might not drastically alter outcomes in commonly used behavioral tests. However, the inflammatory response to stroke, which is integral to PSD pathology, appears to vary between sexes, with females often showing a stronger anti-inflammatory response [275]. Future research should continue to address these variables, providing a deeper understanding of how age and sex influence the development, treatment, and ultimately, recovery from PSD. Such insights are vital for designing tailored neuro- or psychotherapeutic clinical approaches.

Behavioral tests play a crucial role in assessing depressive-like behaviors in animal models that aid our understanding of the multifaceted nature of PSD. These tests evaluate a range of symptoms, from mood disturbances and cognitive deficits to motor dysfunctions and social behavior changes. Commonly employed tests include the sucrose preference test for anhedonia, the forced swim and tail suspension tests for despair-like behavior, or social interaction tests for assessing social withdrawal. Additionally, motor function tests also help to gauge the physical aspects of depression, which are often impacted in post-stroke conditions [39]. Each test is designed to measure specific symptoms associated with PSD, providing a comprehensive view of the animal’s emotional and cognitive state post-stroke. However, interpreting the results of these behavioral tests requires careful consideration and interpretation. The inherent variability in rodent behavior, the subjective nature of depressive symptoms, and the potential for human error in collecting data during the experiment or subsequent analyses necessitate a cautious approach [33,252]. Variations in test conditions, handling, and even the environment can influence the outcomes, potentially affecting the accuracy of the data [166]. Given these challenges, there is a pressing need for future research to focus on refining existing behavioral tests and also develop more sophisticated and direct methods for assessing PSD. This involves enhancing the objectivity, sensitivity, and specificity of behavioral assays to accurately capture the nuanced manifestations of PSD in animal models. Improvements in test design, execution, and data analysis can lead to more reliable and valid measurements of depressive-like behaviors, facilitating the identification of effective treatments and interventions for PSD. 

In conclusion, this review summarized the complex and multifaceted nature of PSD, emphasizing the significant challenges involved in modeling and evaluating this condition in preclinical trials. Our exploration into the epidemiology, risk factors, underlying mechanisms, and the development of animal models for PSD has underscored the crucial need for advanced, nuanced approaches in preclinical research. Indeed, bridging the gap between animal studies and clinical applications requires focused efforts to refine and develop animal models and sophisticated behavioral assessments that more accurately mirror the human condition and behavior. Enhancing these models and assessments is essential for improving the translation of research findings into the clinic, resulting in more effective diagnostic tools and treatments for PSD. This includes addressing the variability in rodent responses, the challenge of extending the results to human pathology, and the integration of diverse biological, psychological, and social determinants of PSD. Additionally, considerations of age, gender differences, and strain variability among rodents highlight the importance of a custom-tailored approach in understanding and treating PSD. This approach will help ensure that the insights gained in the laboratory can be effectively applied in the clinic, ultimately improving patient outcomes and accelerating recovery. While most previous studies address these aspects individually, our aim was to integrate all recent developments in PSD pathology within both clinical and experimental contexts in order to provide a complete perspective, highlighting the gap between human clinical data and preclinical research.

## 11. Summary

PSD stands as a significant barrier to recovery from stroke and is defined by its complexity stemming from an interplay of physiological, psychological, and social factors. PSD research has expanded our understanding, revealing that risk factors such as age, gender, pre-stroke psychiatric history, and the physical location of the stroke significantly influence the likelihood and severity of PSD. Animal models, particularly those involving rodents, are pivotal for understanding the pathophysiological underpinnings of PSD. Models such as MCAO, BCAO, and various genetic models are utilized to mimic stroke in rodents, enabling the study of depressive-like behaviors subsequent to cerebrovascular insults. Techniques combining MCAO with CUMS or social isolation post-stroke have been particularly insightful and highlight the role of PSD environmental and social stressors. Further, behavioral assessments are crucial in measuring symptoms, like anhedonia, despair, hopelessness, and motor dysfunctions, which help evaluate the efficacy of potential treatments and the validity of the models themselves. Despite these advances, translating findings from animal models to human patients remains challenging. Variability in rodent responses, differences in stroke etiology, and the subjective nature of depressive symptoms complicate the direct application of preclinical results to the clinic. Moreover, the outcomes of these studies underline the need for a personalized approach to treatment, considering individual risk factors such as age, sex, comorbidities, social network, etc. While animal models and behavioral studies have greatly contributed to our understanding of PSD, the relatively limited number of clinical studies compared to the many preclinical studies underscores the complexity of this condition and the existing translational gap. Advancements in these areas are vital for developing targeted psychiatric, neuro-, or psychological interventions, ultimately improving the quality of life and recovery outcomes for stroke survivors worldwide.

## Figures and Tables

**Table 1:** CUMS models of depression according to stressors and duration.

Stressors and Duration													Days of CUMS/Mice Housing
Gaignier F., 2018 [124]	Alterations of the light-dark cycle	Cage tilt 1 h, 2 h, 15 h		Food deprivation overnight 15 h	Illumination at night 15 h		Small cage 1 h, 2 h	Soiled cage overnight 15 h	Paired housing 2 h				21 days Individually housing
Zhang M., 2023 [125]	Exposure to a stroboscope 12 h	Cage tilt 12 h	Traffic noise (70–90 dB) 6 h	Food deprivation 12 h	Illumination at night 12 h			Food and water deprivation 24 h	Crowding: ten mice per cage 12 h	Water deprivation 12 h	Level shaking 15 min		28 days Individually housing
Yan W., 2021 [126]	Ice water swimming 5 min	Cage tilt 5 min	Exposure to an empty bottle 1 h	Food deprivation 24 h	Illumination at night 12 h		Restraint stress 2 h	Soiled cage 24 h	Exposure to a foreign object 24 h	Water deprivation 24 h			28 days Group housing
Wu J., 2021 [128]	Ice water swimming 5 min	Cage tilt 24 h	Foot electric shock twice	Food deprivation 24 h	Continuous illumination 24 h		Restraint stress 2 h	Wet bedding 24 h	Tail-clamp 90 s	Water deprivation 24 h	Cage shaking 15 min		35 days Individually housing
Wang Y.I., 2021 [129]	Ice water swimming 5 min	Cage tilt 24 h		Food deprivation 24 h	Continuous illumination 24 h		Restraint stress 6 h	Wet bedding 24 h		Water deprivation 24 h	Cage shaking 30 min		35 days Individually housing
Wang G., 2019 [130]	Ice water swimming 5 min	Cage tilt 12 h	Plantar electrical stimulation 10 min	Food deprivation 12 h	Continuous illumination 36 h		White noise 12 h	Soiled cage 24 h	Tail nipping 2 min	Water deprivation 12 h	Exposure to a stroboscope 2 h		35 days Group housing
Wen G., 2019 [131]	Exposure to a stroboscope overnight	Cage tilt 4 h	120-dB noise overnight	Food deprivation 24 h	Alterations of the light-dark cycle		Restraint stress 4 h	Wet bedding 4 h		Water deprivation 24 h			42 days Individually housing
Li M., 2014 [132]	Alterations of the light-dark cycle	Cage tilt 12 h	Exposure to an empty bottle 10 min	Food deprivation 24 h	Overnight illumination	White noise 1 h	Overhang 10 min	Soiled cage 24 h	Exposure to a foreign object 12 h	Water deprivation 24 h	Tail pinch 1 min	Oscillation 5 min	42 days Group housing
Xie M., 2022 [133]	No bedding 24 h	Cage tilt 24 h		Food deprivation 24 h	Overnight illumination (twice per week)		Restraint stress 6 h	Wet bedding 24 h	Tail pinching 5 min	Water deprivation 24 h	Cage shaking 15 min		49 days Individually housing
Wassouf Z., 2019 [134]	Switched day/night-cycle 48 h	Cage tilt 2 h		Food deprivation 16 h	Illumination at night 12 h		Restraint stress 1 h		Rat confrontation 30 min	Water deprivation 16 h			56 days Individually housing
Wang Y., 2021 [135]	4 °C exposure 1 h	Cage tilt 12 h		Food deprivation 23 h	Day/night inversion		Restraint stress 1 h	Damp bedding 24 h		Water deprivation 23 h	Cage shaking 30 min		56 days Group housing

## Data Availability

No new data were created or analyzed in this study. Data sharing is not applicable to this article.
